# Supplementary material for: Exploring the views and experiences of frailty and resilience among people experiencing homelessness in Ireland: A qualitative study
Source: PLoS One. 2026 Feb 19;21(2):e0343369. doi: 10.1371/journal.pone.0343369 (PMC12919821; doi:10.1371/journal.pone.0343369)
Supplement: S2 Table — (DOCX) [file pone.0343369.s002.docx]

**S2 Table** – Reflexivity statement of principal investigator (TC) in line with Malterud recommendations [1]

| **“Previous personal and professional experiences”** | TC is a male GP and previously completed GP training with a focus on inclusion health, working in areas of deprivation and with marginalised populations. These experiences fostered his interest in homelessness and health inequality. |
| --- | --- |
| **“Pre-study beliefs about how things are and what is to be investigated”** | TC acknowledges a pre-existing belief that people experiencing homelessness (PEH) are a particularly vulnerable group who face significant stigma and barriers when accessing healthcare. He also views frailty as a useful clinical concept for identifying risk and targeting appropriate interventions within this population. |
| **“Motivation and qualifications for exploration of the field”** | TC is currently undertaking a fellowship and MD focused on the intersection of frailty and homelessness. He has prior experience conducting qualitative research and is motivated by a desire to improve healthcare delivery for underserved populations. |
| **“Theoretical foundations related to education and interests”** | TC approaches the topic from a biopsychosocial perspective, with an interest in how frailty and resilience manifest within the context of homelessness. He is particularly committed to capturing the lived experiences of PEH to inform the development and delivery of patient-centred frailty interventions. |

**References**

[1] Malterud K. Qualitative research: standards, challenges, and guidelines. The lancet. 2001 Aug 11;358(9280):483-8.
