# Supplementary material for: Exploring the views and experiences of frailty and resilience among people experiencing homelessness in Ireland: A qualitative study
Source: PLoS One. 2026 Feb 19;21(2):e0343369. doi: 10.1371/journal.pone.0343369 (PMC12919821; doi:10.1371/journal.pone.0343369)
Supplement: S3 Table — (DOCX) [file pone.0343369.s003.docx]

**S3 Table** – Interview topic guide

| 1. What does frailty mean to you? |
| --- |
| 1. What does resilience mean to you? |
| 1. What impact do you think homelessness has on frailty? |
| 1. What supports might help people who are homeless and frail? |
| 1. Is there anything that I haven’t asked about that you feel is important to share related to our exercise and protein intervention or the discussion today? |
