# Supplementary material for: Exploring the views and experiences of frailty and resilience among people experiencing homelessness in Ireland: A qualitative study
Source: PLoS One. 2026 Feb 19;21(2):e0343369. doi: 10.1371/journal.pone.0343369 (PMC12919821; doi:10.1371/journal.pone.0343369)
Supplement: Attachment — Submitted filename: Response to reviewers updated.docx [file pone.0343369.s005.docx]

**Response to reviewers**

We thank the reviewers for their feedback on our manuscript. We greatly appreciate the time and effort they have taken to provide valuable comments that have strengthened our work. We hope that these revisions address the reviewers’ concerns and result in a manuscript that makes a meaningful contribution to the field.

Changes within the main manuscript have been highlighted in yellow and specific responses to each of the reviewer’s comments are set out below:

| **Reviewer 1** | **Authors’ response** |
| --- | --- |
| I recommend the publication of the paper as it fills a gap in the literature: It explores how pre-frail and frail PEH themselves understand frailty and resilience. (Maybe a small weakness in the sample is that all participants were drawn from a feasibility trial and already engaged with healthcare, creating a potential bias toward individuals who are more health-engaged than the general homeless population). It extends frailty research beyond older adults and clinical populations to a younger, socially marginalized group with high premature frailty. It contributes to homelessness research by linking structural conditions directly to frailty trajectories, so shifting the focus from individual frailty traits toward structural determinants and lived context,. It challenges dominant frailty constructs by demonstrating that psychological and social dimensions are central for PEH, implying that existing frailty tools may not be appropriate. | We thank the reviewer for their supportive feedback. We have updated the limitations section in the discussion to further reflect the sample were participants already engaged in healthcare and how this could create a bias as below:  Participants were recruited from a feasibility trial of an intervention designed to reverse frailty and build resilience, which may have introduced selection bias in the sample. As recruitment occurred within a healthcare-based setting, the sample is likely to over-represent individuals who are already engaged with healthcare services or require regular attendance for therapies like opiate substitution therapy, which may limit generalisability to the wider homeless population. However, recruitment was done during routine healthcare appointments, which means the sample reflects patients in this real-world setting. |
| Reviewer 2 | Authors’ response |
| 1. Stronger problematization of the context: While the introduction provides a solid overview of frailty and homelessness, it could be strengthened by a more explicit problematization of why it is particularly important to analyse frailty in this population. Expanding on the social, structural, and biographical mechanisms that lead to accelerated ageing and vulnerability among people experiencing homelessness would help situate the study more clearly within a critical public health perspective. | Thank you for this helpful suggestion. The introduction has been refined to consider the social, structural, and biographical mechanisms contributing to accelerated ageing and vulnerability in this population as below:  Para 3 addition: This phenomenon has been described as ‘premature’ or ‘accelerated’ ageing [19], whereby biological age exceeds chronological age. This is likely the result of intersecting social and structural disadvantages, alongside homelessness itself, which contribute to sustained exposure to stress, illness, and barriers to timely care, which may cumulatively accelerate health decline in this population. This further heightens vulnerability in this group and diminishes their capacity to respond to health challenges, leading to an earlier onset of frailty.  Para 4 addition: While these models were developed primarily in older housed populations, their application to PEH raises important questions about how social and structural determinants of health are captured within existing frailty constructs.  Para 6 addition: This represents a missed opportunity to embed the voices of PEH in the design and delivery of health and social care services aimed at addressing frailty [34], particularly given the distinct elements through which frailty may have been developed and experienced in this population.  Para 8 addition: In doing so, it situates frailty not solely as a biomedical construct, but as one shaped by social, structural, and biographical forces relevant to public health practice and policy. |
| 2.Descriptive characterization of the sample: I suggest adding an additional descriptive table showing participant characteristics by sex and age groups, both at baseline and follow-up. This would allow readers to better understand the heterogeneity of the sample and contextualize the narratives. Variables such as age, sex, frailty level, substance use, and housing situation could be included. | Thank you for this suggestion. This paper reports a secondary qualitative analysis of a subsample of participants (n = 25) who completed post-intervention interviews, rather than the full feasibility cohort (n = 108), for which baseline and follow-up characteristics are reported in the primary trial publication (<https://doi.org/10.3399/BJGPO.2025.0271>). The results section has been revised to include a table of participant characteristics and presented the aggregated descriptive of age, sex, frailty level, accommodation status, opioid agonist therapy, and recent substance use as follows:  Results Para 2 addition: The included participants reflected heterogeneity across age, gender, frailty level, substance use, and housing circumstances (Table 1), providing a diverse cohort from which to explore participants’ views. Participants ranged in age (28-60) and included both pre-frail and frail individuals, with varied experiences of homelessness spanning hostel accommodation, rough sleeping, and insecure housing. High levels of substance use and prescription of OAT were observed, reflecting the complex health and social profiles of the cohort. |
| 3.Deeper analytical use of the narratives: The results section is coherent and well organized, but the analysis could be strengthened by more systematically exploring similarities and divergences across participants, particularly: By age groups (younger vs. older participants). By sex. This comparative perspective could reveal meaningful patterns in how frailty and resilience are experienced and interpreted. | Thank you for this suggestion. The themes identified were broadly shared across participants of different ages and sexes, and contextual factors appear to may have more influenced views. We agree that comparative analysis can enrich qualitative research; however, in this secondary, phenomenologically informed analysis, interviews were not designed to explore age- or sex-based differences. In addition, the modest sample size, particularly the small number of female participants (n = 6) also limits subgroup comparisons. We have therefore clarified in the Results that the themes identified were shared across participants:  Results Para 3 addition: While participants varied in age and sex, the identified themes were broadly shared across the sample, suggesting that experiences were shaped more strongly by homelessness and structural adversity than other factors such as age or gender. |
| 4. Linking narratives to life trajectories and explanatory factors: The discussion could benefit from a more interpretive reflection on the factors potentially associated with the observed narratives (e.g., life course experiences, duration of homelessness, access to institutional support, exposure to violence, mental health, substance use). This would enhance the theoretical contribution of the study and increase its relevance for intervention design and policy. | Thank you for your comment. We have strengthened the discussion to provide a more explicit interpretive reflection on the factors that may underlie participants’ narratives, including cumulative life course adversity, duration and conditions of homelessness, exposure to violence, access to institutional support, mental health, and substance use as follows:  Addition to Principal findings  Para 1: These accounts suggest that frailty among PEH may be influenced by cumulative life course exposures, including prolonged housing instability, adverse childhood events, repeated trauma, and disrupted access to institutional support, rather than chronological ageing alone.  Para 2: These narratives suggested possible biographical pathways into frailty, where early-life adversity, inadequately treated mental illness, and prolonged substance use may interact with homelessness to accelerate vulnerability and functional decline.  Addition to Implications for policy, practice and future research  Para 1: Interventions that fail to consider lack of access to institutional support, exposure to violence, mental illness, substance use and duration of homelessness in this group may have limited effectiveness. |
